# Structure and Sialyllactose Binding of the Carboxy-Terminal Head Domain of the Fibre from a Siadenovirus, Turkey Adenovirus 3

**DOI:** 10.1371/journal.pone.0139339

**Published:** 2015-09-29

**Authors:** Abhimanyu K. Singh, M. Álvaro Berbís, Mónika Z. Ballmann, Michelle Kilcoyne, Margarita Menéndez, Thanh H. Nguyen, Lokesh Joshi, F. Javier Cañada, Jesús Jiménez-Barbero, Mária Benkő, Balázs Harrach, Mark J. van Raaij

**Affiliations:** 1 Departamento de Estructura de Macromoléculas, Centro Nacional de Biotecnología (CNB-CSIC), Madrid, Spain; 2 Departamento de Biología Física-Química, Centro de Investigaciones Biológicas (CIB-CSIC), Madrid, Spain; 3 Institute for Veterinary Medical Research, Centre for Agricultural Research, Hungarian Academy of Sciences, Budapest, Hungary; 4 Glycoscience Group, National Centre for Biomedical Engineering Science, National University of Ireland Galway, Galway, Ireland; 5 Microbiology, School of Natural Sciences, National University of Ireland Galway, Galway, Ireland; 6 Departamento de Química Física-Biológica, Instituto de Química Física Rocasolano (IQFR-CSIC) and CIBER de Enfermedades Respiratorias (CIBERES), calle Serrano 119, E-28006 Madrid, Spain; 7 Centro de Investigación Cooperativa en Biociencias (CIC bioGUNE), Parque Tecnológico de Bizkaia, Derio, Spain; 8 Ikerbasque, Basque Foundation for Science, Bilbao, Spain; University of Roskilde, DENMARK

## Abstract

The virulent form of turkey adenovirus 3 (TAdV-3), also known as turkey hemorrhagic enteritis virus (THEV), is an economically important poultry pathogen, while the avirulent form is used as a vaccine. TAdV-3 belongs to the genus *Siadenovirus*. The carboxy-terminal region of its fibre does not have significant sequence similarity to any other adenovirus fibre heads of known structure. Two amino acid sequence differences between virulent and avirulent TAdV-3 map on the fibre head: where virulent TAdV-3 contains Ile354 and Thr376, avirulent TAdV-3 contains Met354 and Met376. We determined the crystal structures of the trimeric virulent and avirulent TAdV-3 fibre head domains at 2.2 Å resolution. Each monomer contains a beta-sandwich, which, surprisingly, resembles reovirus fibre head more than other adenovirus fibres, although the ABCJ-GHID topology is conserved in all. A beta-hairpin insertion in the C-strand of each trimer subunit embraces its neighbouring monomer. The avirulent and virulent TAdV-3 fibre heads are identical apart from the exact orientation of the beta-hairpin insertion. *In vitro*, sialyllactose was identified as a ligand by glycan microarray analysis, nuclear magnetic resonance spectroscopy, and crystallography. Its dissociation constant was measured to be in the mM range by isothermal titration calorimetry. The ligand binds to the side of the fibre head, involving amino acids Glu392, Thr419, Val420, Lys421, Asn422, and Gly423 binding to the sialic acid group. It binds slightly more strongly to the avirulent form. We propose that, *in vivo*, the TAdV-3 fibre may bind a sialic acid-containing cell surface component.

## Introduction

Adenoviruses have a non-segmented, linear, double-stranded DNA genome packed into non-enveloped virions, icosahedral in shape and around 90 nm in diameter. Based on phylogeny and genome organization, the *Adenoviridae* family is split into five genera accepted by the International Committee on Taxonomy of Viruses (http://www.ictvonline.org): *Mastadenovirus*, *Aviadenovirus*, *Atadenovirus*, *Siadenovirus* and *Ichtadenovirus* [1]. Mastadenoviruses infect mammalian species, including humans, while aviadenoviruses infect birds exclusively. The remaining three genera have been established more recently. Atadenoviruses have been isolated from squamate reptile hosts, ruminants, birds and from a marsupial [2–5]. The origin of siadenoviruses is not clear and they have been isolated from one amphibian host, from Sulawesi tortoises and from several species of birds [1, 6–8]. The genus takes its name from a specific open reading frame with high sequence similarity to bacterial sialidases [6], although the functional significance of this gene is not known yet.

The facets of the adenovirus capsid are made up of the hexon protein, while the penton base forms the vertices. A trimeric fibre is inserted into each vertex. Normally there is one fibre trimer at every vertex, but in the case of fowl adenoviruses two different fibres are incorporated into each penton base [9]; and in some pentons of the lizard adenovirus 2 three identical fibres are present [10]. Besides these, there are some minor structural proteins, which are necessary for assembly and function as capsid stabilizers [11]. The fibre protein interacts with the host cell through its distal, carboxy-terminal, head or knob domain. Receptor-binding and cell entry have been studied almost exclusively in human mastadenoviruses. Depending on the human mastadenovirus species, the fibre head may recognize one or more cell surface receptors such as the coxsackievirus and adenovirus receptor [12], desmoglein 2 [13], CD46/80/86 [14, 15] and sialic acid [16]. This primary interaction is followed by a secondary interaction involving the penton base and cell surface integrins, promoting viral internalization via endocytosis [17].

Human and animal adenoviruses may be useful as vectors for gene and cancer therapy, and may also serve as vaccination agents [18–21]. Adenoviruses from animal hosts may have some advantage for this purpose, due to the expected absence of a pre-existing immunity in humans. Given the implication of fibre head domains in receptor interaction, their structures may inform about the tropism of the viruses and ultimately aid the design of gene therapy vectors, anti-cancer biologics and vaccination vehicles [22, 23]. The structure of the head domain of canine adenovirus 2 fibre [24] has been published, as well as that of both fibres of fowl adenovirus 1 [25, 26]. Structural studies of the carboxy-terminal end of a porcine adenovirus 4 fibre have shown that apart from a canonical head domain, it contains a unique tandem carbohydrate-recognizing galectin domain [27]. The high-resolution structure of an atadenovirus fibre head (snake adenovirus 1) has recently been reported and is the smallest fibre head domain solved so far [28]. In this fibre head, the topology of the beta-sandwich is conserved, but most of the loops connecting the beta-strands are shorter than in previously solved structures and the DG-loop contains an alpha-helix.

Known siadenovirus species are *Frog siadenovirus A* (FrAdV-A), *Raptor siadenovirus A* (RAdV-A), *Great tit siadenovirus A* (GTAdV-A) [29], and *Turkey siadenovirus A* (TAdV-A). The latter currently contains as a sole member turkey adenovirus 3 (TAdV-3), which is also named turkey hemorrhagic enteritis virus (THEV) [1]. A fifth species, *Skua siadenovirus A*, has been discovered more recently [8]. Several further adenoviruses detected in avian hosts also belong to the genus *Siadenovirus*, including psittacine adenovirus 2 [30, 31] and Gouldian finch adenovirus 1 [32]. A siadenovirus was also recovered from captive Sulawesi tortoises [33]. Siadenoviruses possess the shortest genomes in the *Adenoviridae* family (about 26 kb). Besides the well-conserved central gene cassette, only six additional, genus-specific open reading frames have been described [1, 6–8, 34, 35].

Virulent TAdV-3 strains can cause hemorrhagic enteritis in turkeys, which is often fatal, splenomegaly in chickens and marble spleen disease in pheasants [36–38]. The less virulent strains are suitable vaccines against turkey hemorrhagic enteritis [39–42]. A comparison of the genome sequences of a virulent [35] and an avirulent TAdV-3 strain has revealed eight mutations, of which four do not cause a corresponding amino acid change, one maps to the putative sialidase gene, one to a potential transcriptional regulator (E3), and two map to the fibre gene [43]. The TAdV-3 fibre protein has 454 residues, of which 1–45 are probably the tail domain that binds to the penton base, 46–303 form a putative shaft domain with fifteen triple beta-spiral sequence repeats [44] and 304–454 may form the head domain [45]. Here we describe the crystallographic structure of this carboxy-terminal end of the TAdV-3 fibre and confirm it as a globular, trimeric head domain which, surprisingly, most closely resembles the structure of reovirus fibre heads. We compare the structures of the virulent and avirulent fibre heads, and show that, in both cases, the head domain can bind sialyllactose.

## Materials and Methods

### Protein production, crystallization and data collection

Construction of the expression vector for the avirulent version of the TAdV-3 fibre head domain (residues 304–454) and protein expression, purification and crystallization of this protein and the selenomethionine derivative of it has been described [45]. The protein expressed from this vector (called pET28a(+)-THEVfib304-454) has an amino-terminal purification tag (M GSSHH HHHHS SGLVP RGSHM ASMTG GQQMG RGSEF).

To prepare the virulent version of the fibre head protein, the codons for Met354 and Met376 in the same expression plasmid were modified to Ile and Thr, respectively, using a two-step site-directed mutagenesis procedure. In the first step, a 50 μl reaction mixture was prepared containing 200 ng of the plasmid construct pET28a(+)-THEVfib304-454, complementary oligonucleotides containing the M354I mutation and NZYDNA-Change polymerase (NZYTech, Lisbon, Portugal), which was then subjected to 18 cycles of PCR with a final extension step of 7 min. Post amplification, the PCR product was treated with 40 units of DpnI (Agilent Technologies, Madrid, Spain) for 3 h at 37°C in order to digest parental plasmid DNA. The introduced mutation was confirmed by DNA sequencing (Secugen, Madrid, Spain) and the plasmid named pET28a(+)-THEVfib304-454mut354. The mutagenesis procedure was repeated with pET28a(+)-THEVfib304-454mut354 to introduce the Met376Thr mutation. The resulting plasmid was named pET28a(+)-THEVfib304-454vir. Single amino acid mutants for the avirulent version fibre protein (Arg368Ala, Lys439Ala, Asn407Ala, Lys421Ala and Glu392Ala) were generated using the same procedure, but in a single step. They were named pET28a(+)-THEVfib304-454mut368, pET28a(+)-THEVfib304-454mut439, pET28a(+)-THEVfib304-454mut407, pET28a(+)-THEVfib304-454mut421 and pET28a(+)-THEVfib304-454mut392 respectively. All plasmids were checked by DNA sequence analysis (Secugen, Madrid, Spain).

Expression and purification of the virulent fibre head and the five point-mutants were carried out in the same way as described for the native avirulent fibre head protein [45]. The virulent fibre head protein was desalted and concentrated to 10 mg/ml in 25 mM MES [2-(*N*-morpholino)ethanesulfonic acid]–NaOH pH 6.0 using Amicon Ultra concentrators (Millipore Iberica, Madrid, Spain) and stored temporarily at 4°C prior to use in crystallization trials, whereas the rest of the mutant proteins were concentrated to between 7 and 12 mg/ml to be used for the biophysical analyses described below. Crystallization trials for virulent fibre head protein were performed by sitting drop vapour diffusion. Cube-shaped crystals, very similar to the avirulent version, were obtained in a period of 2–3 days when the crystallization solution contained 1 M diammonium phosphate and 0.1 M imidazole, pH 7.0. Crystals were soaked in growth solution supplemented with 25% glycerol and mounted in LithoLoops (Molecular Dimensions, Newmarket, Suffolk, England) or MicroMounts (Mitegen, Ithaca, New York, USA) prior to vitrification in liquid nitrogen. Ligand soaking experiments of TAdV-3 fibre head crystals were carried out at 21°C for at least two hours, maintaining a concentration of between 30 and 50 mM of 3'- or 6'-sialyllactose (Elicityl, Crolles, France) in crystallization solution. After soaking, crystals were transferred to the same ligand-containing crystallization solution supplemented with 25% glycerol and mounted for data collection.

### Structure solution

Crystallographic data collection for the avirulent version of the TAdV-3 fibre head domain (residues 304–454) and the selenomethionine derivative has been described [45]. X-ray diffraction data for the virulent version fibre head crystals were collected at beamline XALOC-BL13 of the ALBA Synchrotron Light Facility (Barcelona, Spain) using a Pilatus 6M pixel detector (Dectris Ltd., Baden, Switzerland), and for the ligand-bound forms at BM30 of the European Synchrotron Radiation Facility (Grenoble, France) using a Q315r CCD detector (ADSC, Poway CA, USA). All data sets are of space group *I*23. Crystallographic data were integrated using IMOSFLM [46] and reduced using POINTLESS, SCALA and TRUNCATE [47], all integrated in the Collaborative Computational Project Number 4 [48]. Structure solution of the selenomethionine version of the avirulent form fibre head was performed with the AUTOSHARP pipeline [49], which incorporates SHELX [50] for locating heavy atom sites, SOLOMON [51] for solvent correction and ARP-WARP for automated density interpretation [52]. Adjustment of the model was performed with COOT [53] and refinement with REFMAC5 [54]. Reflections for calculating the free R-factor were selected randomly. The same reflections were selected for the free and working R-factor for refining the selenomethionine-derivatised avirulent version, the avirulent version, the virulent version and the ligand-bound models. Validation was carried out with MOLPROBITY [55]; interaction properties, including buried surface area, were calculated with PISA [56] as implemented by the European node of the Protein Structure Database (http://www.ebi.ac.uk/msd-srv/prot_int/cgi-bin/piserver). Structural superpositions, using secondary structure element matching, were carried out with CCP4MG [57]. Structure similarity searches were performed with DALI [58]. Structure figures were made with PYMOL (Schrödinger LLC, Cambridge MA, U.S.A.).

### Thermofluor protein unfolding assay

Thermal shift assays [59] were carried out in an iCycler iQ PCR Thermal Cycler (Bio-Rad, Hercules CA, USA) in the presence of the fluorescent dye SYPRO Orange (Life Technologies SA, Madrid, Spain). Reaction volumes of 30 μL were prepared in 200 μL capped PCR tubes with 30 μg of protein and 1X SYPRO Orange from the supplied 5000X stock solution. Thermal denaturation curves were obtained by heating the samples from 4°C to 94°C with a ramp rate of 1°C/min and monitoring the fluorescence at every 0.5°C increment. The melting temperature Tm is defined as the point where the slope of the fluorescence increase is maximal.

### Glycan microarray binding assays

The proteins were profiled on neoglycoconjugate microarrays to identify potential glycan ligands for TAdV-3 fibre head proteins. The microarrays were constructed as previously described and presented 71 different simple to complex glycans on glycoproteins or attached to protein backbones through linkers (S1 Table) [60, 61]. Several viral protein concentrations were titrated in Tris-buffered saline supplemented with Ca^2+^ and Mg^2+^ ions (TBS; 20 mM Tris-HCl, 100 mM NaCl, 1 mM CaCl_2,_ 1 mM MgCl_2_, pH 7.2) and with 0.05% Tween 20 (TBS-T). It was determined that 10 μg/ml in TBS-T was the concentration for optimal signal to noise ratio. Similarly, the concentration of the anti-6XHis IgG-CF640R (Sigma Aldrich, Tres Cantos, Spain) monoclonal antibody for detection of viral protein binding was optimized and a 1 in 750 dilution in TBS-T was subsequently used.

Two-step incubations were carried out as previously described with minor modifications [61]. First, the viral proteins were incubated on the microarray slides for 1 h at 23°C and 4 rpm. The microarray slides and gaskets were then separated while submerged under TBS-T, washed 3 times in TBS-T for 2 min each with gentle agitation in a Coplin jar, with a final 2 min wash in TBS. After centrifuging to dry the slides (1,500 rpm, 5 min), all subsequent steps were carried out in the dark. The microarray slides were incubated with the fluorescently labelled anti-6XHis IgG as in the first step, washed and dried by centrifugation and scanned immediately on an Agilent G2505 microarray scanner using the Cy5 channel (633 nm excitation, 80% PMT, 5 mm resolution). All experiments were carried out in triplicate and binding inhibition assays were performed in parallel in the presence of 100 mM galactose and lactose to verify specific binding [62]. Data extraction and analysis was carried out as previously described [60, 61]. Binding was taken as above an experimental threshold of five times the background, which was approximately 1,600 relative fluorescence units (RFU) in this case [60, 63].

### Saturation transfer difference NMR spectroscopy

Saturation transfer difference nuclear magnetic resonance spectroscopy (STD-NMR) experiments were performed at 298 K in an AVANCE 500 MHz spectrometer (Bruker, Rivas Vaciamadrid, Spain) equipped with a 5 mm inverse probe head. STD experiments were performed in fully deuterated 10 mM potassium phosphate pH 7.7. Samples contained 1.5 mM of either 3'- or 6'-sialyllactose and 15 μM of either the virulent or the avirulent versions of TAdV-3 fibre head. The protein was saturated on-resonance at 0.5 ppm and off-resonance at 100 ppm with a train of Gaussian-shaped pulses of 50 ms each, totalling an irradiation time of 2 s. A T_2_ relaxation filter consisting of a 15 ms 5 kHz spin-lock was used to reduce the protein background signals. STD spectra were obtained by subtracting the on-resonance spectra from the off-resonance spectra. STD intensities were measured comparing each STD spectrum with the correspondent off-resonance spectrum and normalizing to the ligand peak receiving the highest degree of saturation (in all cases, the 5''-*N*-acetyl). The resonances of 3'- or 6'-sialyllactose were assigned by standard procedures (^1^H-^1^H TOCSY 70 ms, ^1^H-^1^H ROESY 600 ms and phase-sensitive ^1^H-^13^C HSQC). Data were analyzed with TOPSPIN (Bruker) and figures were made with MESTRENOVA (Mestrelab Research, Santiago de Compostela, Spain).

### Isothermal titration calorimetry

Isothermal titration calorimetry (ITC) was performed at 25°C with a Microcal VP-ITC microcalorimeter (GE Healthcare, Madrid, Spain). The proteins were exhaustively dialyzed against the appropriate buffer (10 mM MES-NaOH pH 6.0 or 20 mM Tris-HCl, 100 mM NaCl pH 7.2), and sialyllactose solutions were prepared in the final dialysate. Titrations were performed by stepwise injections of the 3'- or 6'-sialyllactose-containing solution (21–40 mM) into the reaction cell loaded with the protein at concentrations of 78–474 μM. When required, a second set of injections followed the first one after refilling the injection pipette with the same sialyllactose solution. The CONCAT32 (GE Healthcare) program was used to concatenate the raw data of both series. The heat of ligand dilution was determined separately and subtracted from the total heat produced following each injection. Protein concentrations were determined spectrophotometrically at 280 nm using the predicted monomeric molar extinction coefficient (10,095 OD cm^-1^ M^-1^). Titration data were analyzed with the ORIGIN software (GE Healthcare).

## Results and Discussion

Based on the absence of triple beta-spiral shaft repeat sequences and their location at the carboxy-terminus of the TAdV-3 fibre, amino acids 304–454 are expected to form a fibre head domain, although they do not show appreciable sequence similarity with other adenovirus head domains [45]. We previously reported the construction of an expression vector for the putative fibre head domain fused to an amino-terminal purification tag containing six consecutive histidine residues, which was used to express the protein and followed by purification and crystallization [45]. A complete and highly redundant X-ray diffraction data set was collected on a crystal grown from protein derivatised with selenomethionine, at a wavelength at which the anomalous signal of the selenium atoms was maximized [45]. The data used extended to 2.2 Å resolution.

Out of six selenium sites expected from the sequence, four were located by the automated phasing procedure, and refinement of these sites resulted in high-quality phases (see phasing statistics in Table 1). Solvent flattening resulted in an easily interpretable map in which 137 amino acids were automatically traced. The asymmetric unit of the crystal contains one protein monomer and the biologically relevant trimer is generated by the crystallographic three-fold symmetry axis. Manual completion resulted in a model consisting of 138 amino acid residues (317–454), one phosphate and 47 water molecules. The model was also independently refined against data collected from crystals of the native, non-selenomethionine-containing avirulent and virulent forms, and here manual adjustment resulted in a model containing the same 138 residues plus ordered solvent (Table 1). The phosphate ion mediates a crystal contact between Lys421 and Asn422 of one monomer and Arg368 and Lys439 of a monomer belonging to another trimer. No density was visible for residues amino-terminal to 317 or for the purification tag.

**Table 1:** Crystallographic data and refinement statistics for native and selenomethionine derivative TAdV-3 fiber head crystals.

	avirulent selenomethionine	avirulent native	virulent native	avirulent with 3'-sialyllactose	avirulent with 6'-sialyllactose
**Data collection**					
Wavelength (Å)	0.9791	0.9768	0.9795	0.9797	0.9795
Space group	*I*23	*I*23	*I*23	*I*23	*I*23
Cell edge (Å, a = b = c)	98.75	98.57	98.96	98.31	98.46
Resolution range (Å)	30–2.2 (2.32–2.20)	30–2.2 (2.32–2.20)	31.3–2.3 (2.42–2.30)	25–2.5 (2.64–2.5)	25–2.2 (2.32–2.20)
Reflections	8297 (1201)	8256 (1183)	7327 (1053)	5625 (811)	8219 (1177)
Multiplicity	43.2 (43.1)	11.6 (12.0)	8.9 (9.2)	7.7 (7.9)	9.3 (9.6)
Completeness (%)	100.0 (100.0)	100.0 (100.0)	100.0 (100.0)	100.0 (100.0)	99.9 (100.0)
Mean <I/s(I)>	29.0 (6.5)	17.5 (3.8)	6.3 (1.4)	14.6 (2.6)	18.8 (4.0)
R_sym_ (%)	10.4 (81.4)	8.8 (69.4)	6.2 (53.4)	10.0 (96.5)	7.1 (58.0)
Wilson B (Å^2^)	45.1	43.1	56.8	44.6	40.1
**Phasing**					
Heavy atom sites	4 Se				
Correlation coefficient (all/weak)	55.47 / 33.95				
Patterson figure of merit	22.19				
Correlation coefficient (E)	0.500				
R-cullis (anomalous, acentric)	0.574				
Phasing power (anom. differences)	2.066				
FOM	0.4152 / 0.0728				
**Solvent flattening** (44.6% solvent)					
R-factor (before/after)	0.4800 / 0.2760				
Overall corr. on |E|^2^ (before/after)	0.3057 / 0.7166				
Corr. on |E|^2^ / contrast (orig./inv.)	0.4630 / 0.2240				
**Refinement**					
Reflections used	7909 (1145)	7866 (1128)	6962 (984)	5355 (768)	7836 (1121)
Reflections used for *R*_free_	385 (56)	384 (55)	334 (40)	261 (43)	383 (56)
*R*-factor	0.207 (0.254)	0.199 (0.251)	0.197 (0.245)	0.193 (0.28)	0.177 (0.22)
*R*-free	0.252 (0.295)	0.242 (0.332)	0.239 (0.321)	0.254 (0.34)	0.215 (0.32)
Protein/phosphate/water atoms	1092/5/47	1092/5/57	1091/10/40	1092/21/18	1092/21/60
<B> protein/ligand/water (Å^2^)	41.9/74.9/43.3	41.7/83.8/43.7	65.4/59.3/52.8	53.1/68.0^a^/33.4	47.7/56.1^b^/46.8
Ramachandran statistics (%)^c^	96.3/99.3	97.1/99.3	94.1/99.3	94.1/99.3	96.3/99.3
r.m.s.d. bonds (Å)/angles (°)	0.014/1.5	0.014/1.5	0.013/1.5	0.012/1.7	0.012/1.6
PDB code	3ZPF	3ZPE	4CW8	4D62	4D63

Values in brackets are for the highest resolution bin, where applicable.

^a^Refined with an occupancy of 1.00.

^b^Refined with an occupancy of 0.67.

^c^Favourable/allowed.

The final refined structures have good geometry and acceptable Ramachandran statistics (Table 1). The structures each contain one clear *cis*-peptide bond, between Ser374 and Pro375. The head domain appears to start at around residue 317, rather than around 304 as predicted. It is possible that the shaft domain ends around residue 303, and the head domain, starting at residue 317, is connected to the shaft domain by a short linker sequence as in human adenovirus 2 (HAdV-2; [44]), where it confers flexibility between the head and shaft domains. Studies on larger fragments or the whole fibre would be necessary to show if this is also true for the TAdV-3 fibre.

### Structure of the fibre head

**Fig 1:**
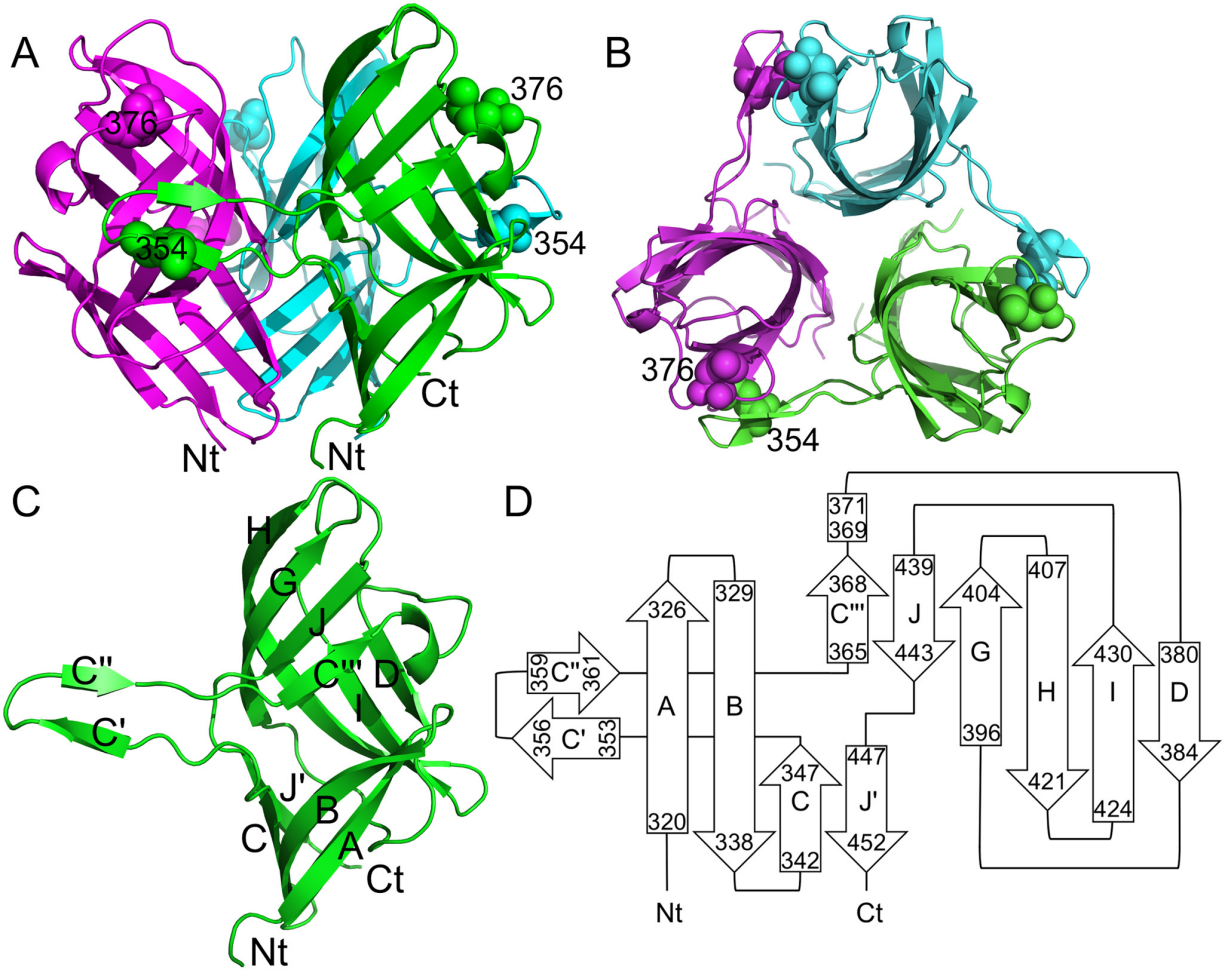
Structure of the TAdV-3 fibre head. A) Side view of the fibre head trimer. B) Top view of the fibre head trimer. Between panels A and B the structure has been turned approximately 90° towards the reader. Each monomer is coloured differently. C) Side view of a monomer with the amino-terminus, carboxy-terminus and beta-strands labelled. D) Topology diagram of the monomer labelled as in panel C, but with the start and end residue of each strand also indicated. In panels A and B, Met354 and Met376 are shown in space-filled representation and labelled.

The TAdV-3 fibre head is around 5 nm high and 4.5 nm in diameter (Fig 1). The topology is similar to that reported for other adenovirus fibre head structures [64, 65], despite the low sequence identity (only 10–20% sequence identity when aligned with known adenovirus head domain structures). Each monomer contains an eight-stranded beta-sheet (ABCJGHID), in which the C- and J-strands are kinked (Fig 1D). The C-strand is interrupted by a 27-residue stretch that forms a beta-hairpin "arm" contacting a neighbouring monomer (see below). We named the bottom section of the C-strand C, the beta-strands of the arm C' and C'', and the top section of the C-strand C‴ (Fig 1). Similarly, the J-strand is divided into an upper (J) and a lower part (J'). The upper part (J) interacts both with the C‴-strand and the G-strand, while the lower part (J') interacts with the C-strand. Beta-strands E and F present in the HAdV-5 fibre head [64] are absent in our structure. The beta-strands in the fibre head are predominantly connected with short loops. The C‴D-loop contains a few residues (369–371) in 3_10_-helical conformation. The long DG-loop runs along the bottom of the beta-sandwich and contains amino acid side chains that contribute significantly to the hydrophobic core of the monomer. The insertion of a beta-hairpin in the C-strand contacting a neighbouring monomer is a unique feature of this structure. Out of the sixteen residues of the beta-hairpin, eleven participate in inter-monomer contacts.

### Trimer stability

**Fig 2:**
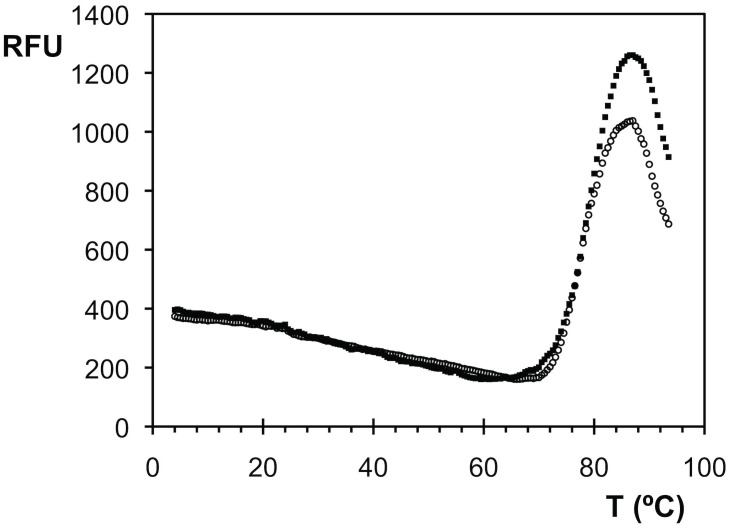
Thermal stability assay of virulent and avirulent TAdV-3 fibre head proteins. The relative fluorescence emission intensity (RFU, arbitrary unit) is plotted as a function of the temperature. Data for virulent (open circles) and avirulent (filled squares) TAdV-3 fibre head proteins are shown. A melting temperature of around 80°C was estimated for both proteins.

Each TAdV-3 fibre head monomer has a surface area of 9.4 x 10^3^ Å^2^, of which 2.6 x 10^3^ Å^2^ (27%) is buried in the trimer. The calculated energy released upon trimer formation is around 60 kcal/mol and a salt bridge is formed between Glu401 and Arg390 of a neighbouring monomer. These properties resemble those of other adenovirus and reovirus fibre heads, suggesting that the stability of the trimer is comparable. As for other trimeric fibre heads, we observed trimers in denaturing gel electrophoresis if the sample was not previously boiled [66]. The melting temperatures of both the virulent and avirulent forms of the protein are 80°C at pH 6 (Fig 2), which indicates the protein is very stable [67].

### Differences between the avirulent and virulent fibre head

**Fig 3:**
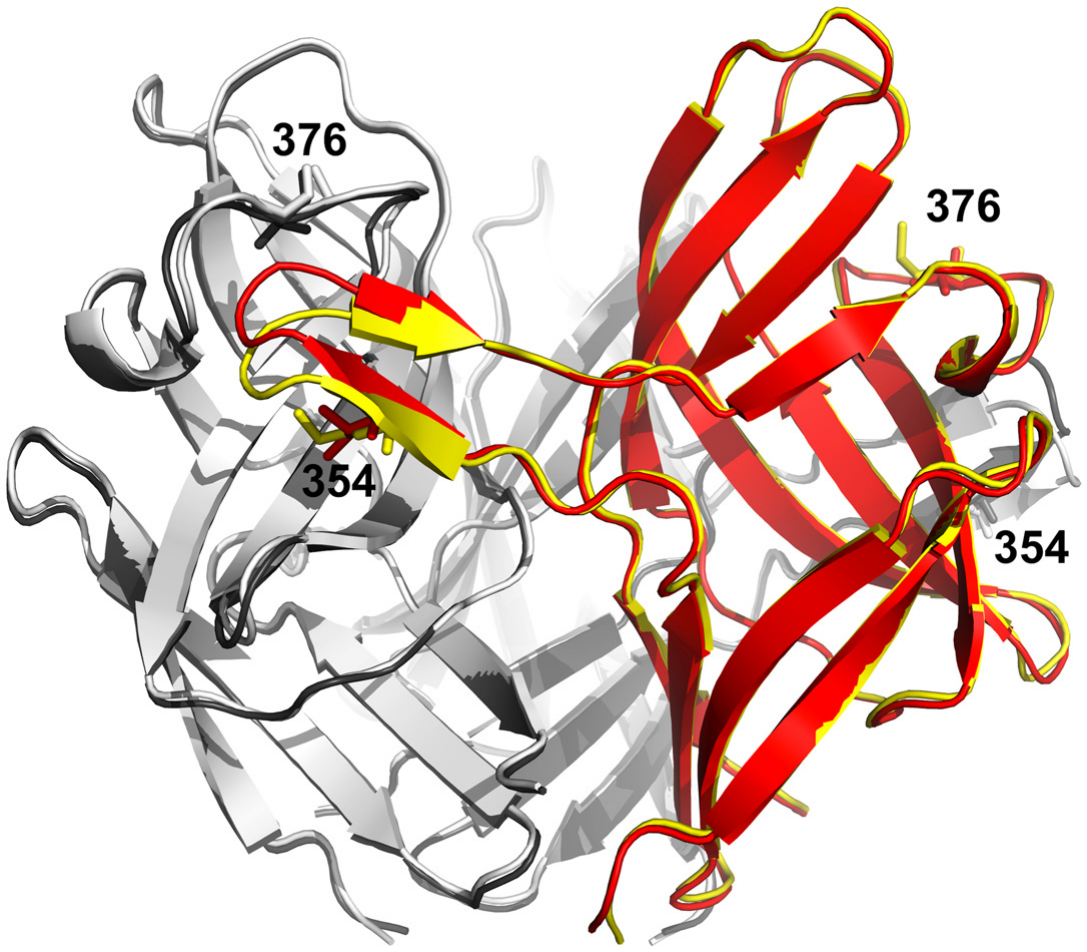
Comparison of the structures of the fibre head trimers of the virulent and avirulent variants of TAdV-3. One monomer of the virulent form is shown in red secondary structure cartoon representation, the other two in gray, while the avirulent fibre head trimer is shown in yellow and white, respectively. Residues Ile354 and Thr376 of the virulent form and Met354 and Met376 of the avirulent form are shown in stick representation and labelled.

The structures of avirulent and virulent forms are virtually identical (root mean squared difference (r.m.s.d.) of 0.53 Å when the C-alpha atoms of residues 317–454 are superposed). The largest difference is in the C'C"-loop, which moves upwards by up to 3 Å (Fig 3). This loop is near the two residues that differ between the avirulent and virulent forms, i.e. near residue 354 located in the C'-strand (Met in the avirulent form and Ile in the virulent form), but also near in space to residue 376 in the C"D-loop of a neighbouring monomer, which is Met in the avirulent form and Thr in the virulent form. The different position of the C'C"-loop can perhaps at least in part be attributed to the side chain of residue Ile354, which appears to push it further away.

### Comparison with other fibre heads

**Fig 4:**
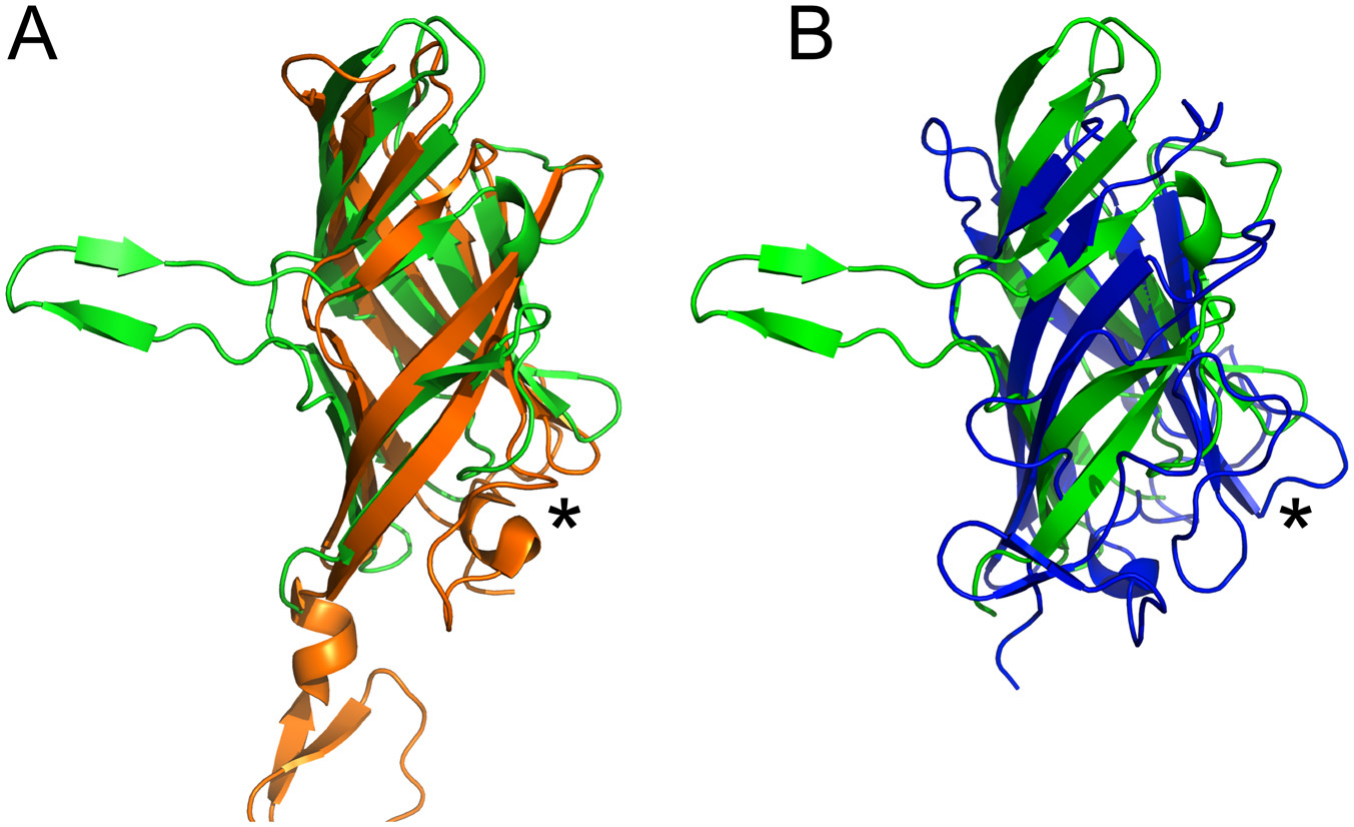
Superposition of the TAdV-3 fibre head monomer onto the structural homologues. (A) Superposition of the TAdV-3 fibre head monomer onto the mammalian reovirus sigma1 head domain and the porcine adenovirus 4 NADC-1 strain fibre head domain (B). Superposition of the TAdV-3 fibre head monomer onto the mammalian reovirus sigma1 head domain and the porcine adenovirus 4 NADC-1 strain fibre head domain The TAdV-3 fibre head monomer is shown in green, the sigma1 head domain in orange and the porcine adenovirus 4 NADC-1 strain fibre head domain in blue. An asterisk shows the location of the DG-loops, it can be seen that the DG-loop of the TAdV-3 fibre head is shorter than its reovirus sigma 1 and porcine adenovirus 4 NADC-1 strain fibre head counterparts.

A structure similarity search resulted in multiple hits. Curiously, the TAdV-3 fibre head monomer topology is more similar to reovirus fibre heads sigma1 and sigmaC [68, 69] than to other adenovirus fibre heads (Fig 4). When our structure is superposed on the mammalian reovirus sigma1 fibre head domain, PDB entry 2OJ5 (Fig 4A) [70], the r.m.s.d. is 2.9 Å for 106 superimposed C-alpha atoms (Z-score 8.3). The next most similar protein identified is the lactococcal bacteriophage TP901-1 receptor-binding protein, PDB entry 2F0C [71], with an r.m.s.d. of 2.9 Å for 90 superimposed C-alpha atoms (Z-score 5.7). Finally, when the structure is superposed onto the HAdV-5 head domain, PDB entry 1KNB [64], the r.m.s.d. is 3.8 Å for 96 superimposed C-alpha atoms (Z-score 4.8).

The TAdV-3 fibre head shares the secondary structure topology with reovirus fibre heads, and also the kinked nature of strands C and J (the latter is called H in the reovirus fibre heads). Other adenovirus fibre heads do not have kinks in the C-strand and only have the equivalent of the J'-strand and not the J-strand. An evolutionary link has been proposed between adenovirus fibres and reovirus attachment proteins, based on their striking similarity in beta-sheet composition and topology of the head domain and the presence of beta-spiral repeats in their shaft domains [68]. However, the eight anti-parallel beta-strands present in both of these proteins have a somewhat different arrangement, even if the topology is the same. In the case of adenovirus fibres, these anti-parallel sheets are arranged in a beta-sandwich architecture of two four-stranded sheets, while in reovirus sigma1 and sigmaC the eight beta-strands form a beta-barrel, i.e. the two sheets contact each other at the ends [68, 69]. In the case of the TAdV-3 fibre head, the two beta-sheets contact each other only at one end, the one that is located towards the inside of the trimer (Fig 1).

Besides reovirus proteins, the search also revealed the lactococcal phage TP901-1 receptor-binding protein [72] as a structural homologue. Like the TAdV-3 fibre head, the carboxy-terminal head domain of this protein (residues 63 to 163) contains an eight-stranded curved beta-sheet. However, the lactococcal phage protein head domain is much smaller, with most beta-strands being shorter (in other words, the TAdV-3 fibre head is "taller").

When comparing the TAdV-3 fibre head to adenovirus fibre heads only, the closest neighbour is that of the porcine adenovirus 4 [27] (Fig 4B), whereas the closest HAdV analogues are the fibre head of HAdV-19p and HAdV-37, which may bind both the coxsackievirus and adenovirus receptor protein and/or sialic acid [73]. As all other adenovirus fibre heads known, they contain a beta-sandwich of four beta-strands in each sheet, in which the two sheets do not contact each other at any of the sides to form a larger curved sheet or beta-barrel. None of these other trimeric receptor-binding domains show the beta-hairpin insert in the C-strand of the TAdV-3 fibre head. Both reovirus and other adenovirus fibre heads have an elaborate DG-loop, which in both cases is involved in receptor-binding [74, 75]. The TAdV-3 fibre head has a shorter DG-loop (Fig 4).

### Sialyllactose binding

**Fig 5:**
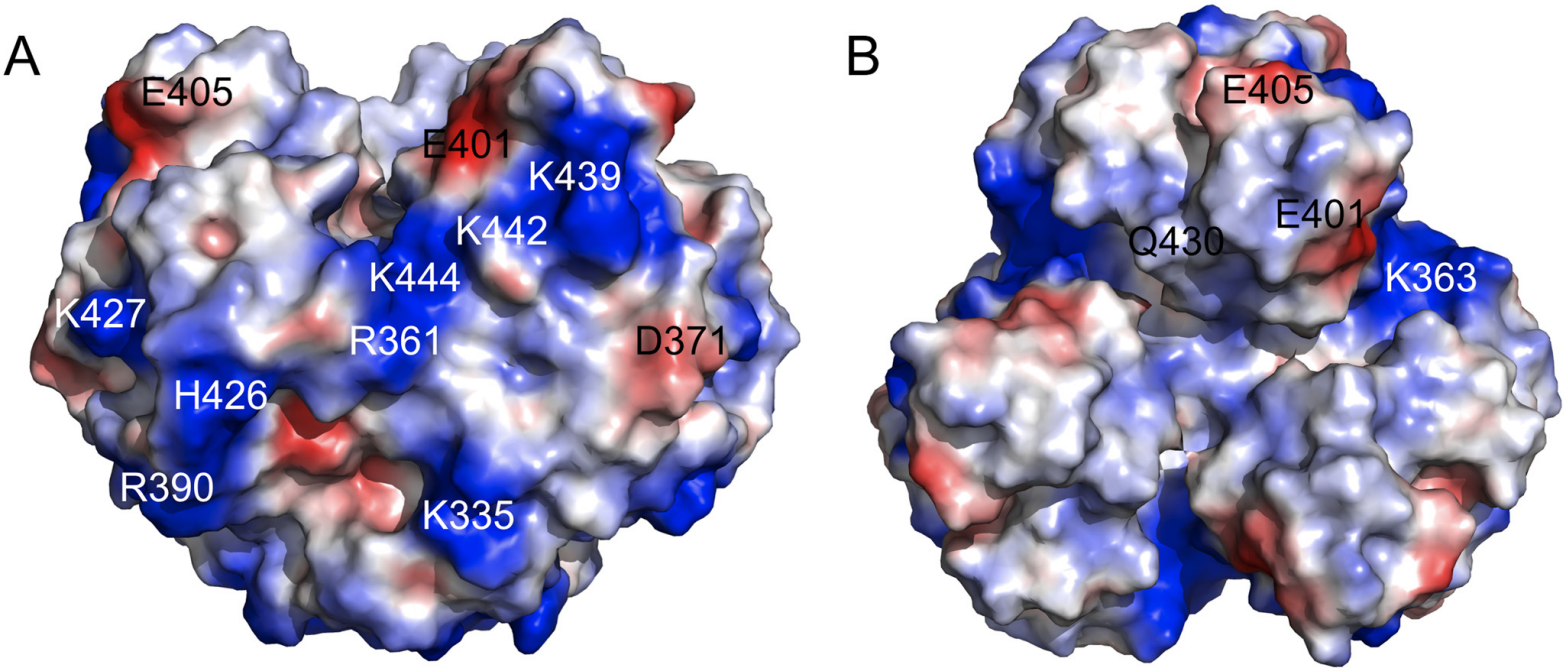
Surface properties of the TAdV-3 fibre head. A) Side view. B) Top view. Calculated negatively charged regions are shown in red and positive regions in blue. The location of some of the polar, electropositive and electronegative amino acid side-chains that contribute to these charged regions are indicated.

Different adenovirus fibre heads have different surface charge distributions and predicted iso-electric point values. HAdV-3 has a predicted iso-electric point of 5.0, while HAdV-37 has a value of 9.1. The TAdV-3 fibre head has an even higher predicted iso-electric point of 9.9 and significant electropositive patches are present on the surface of the trimer (Fig 5). As mentioned earlier, HAdV-37 fibre head binds sialic acid (as well as the coxsackievirus and adenovirus receptor protein), and the interaction with sialic acid is charge dependent [76]. The fact that a siadenovirus-specific open reading frame exists of which the putative gene product has a high sequence similarity with bacterial sialidase proteins also prompted us to perform experiments to try to identify a carbohydrate molecule that might bind the TAdV-3 fibre head.

**Fig 6:**
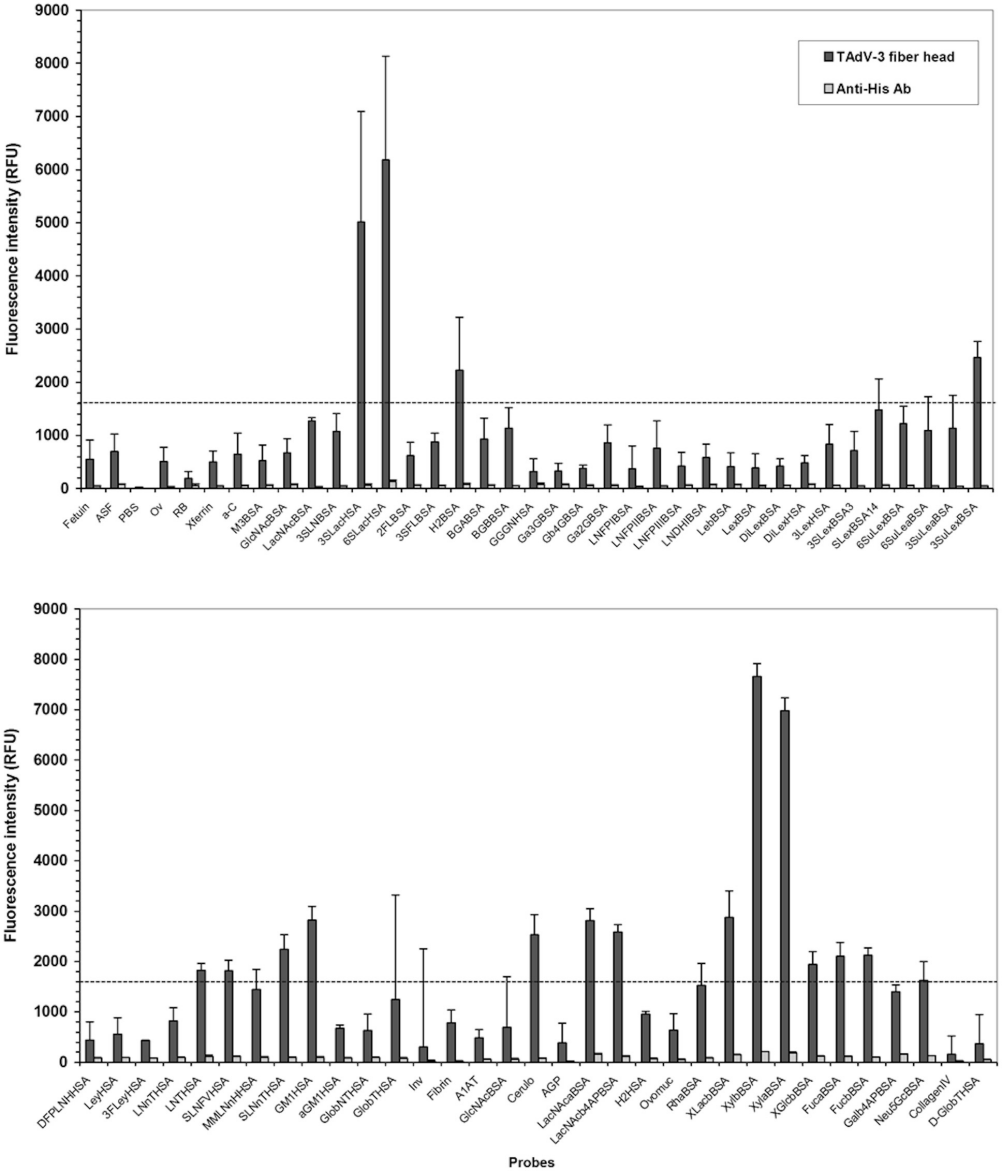
TAdV-3 fibre head binding to a glycan microarray. A histogram representing the fluorescence intensity of TAdV-3 bound to presented glycans on the microarray surface detected by fluorescently-labelled anti-His antibody is shown. Histograms represent the average of three replicate experiments and error bars depict one standard deviation of the mean calculated over three microarray slides. The broken line represents the binding threshold (approximately five times background).

Glycan microarray profiling of the His-tagged TAdV-3 fibre head protein was performed with a library of 71 glycoconjugates (Fig 6; S1 Table). The protein showed substantial interaction with two sialylated glycans, 3'- and 6'-sialyllactose. The observed fluorescence intensities were 5,000 and 6,100 RFU for 3'- and 6'-sialyllactose, respectively, which was significantly higher than the experimental binding threshold of approximately 1,600 RFU (five times background; Fig 6) [60, 63]. No binding inhibition was observed in the presence of lactose or galactose, indicating that the sialic acid portions of the carbohydrates were mainly involved in contact with the proteins. Xylose linked to bovine serum albumin through a 4-aminophenyl linker was also identified as a potential ligand, although the biological significance of this result is doubtful, as terminal xylose units are only found in plant glycans. Interestingly, TAdV-3 did not bind to 3'-sialyl-*N*-acetyllactosamine (3SLNBSA), as is found in *N*-linked oligosaccharides on glycoproteins, or to glycoproteins including bovine fetuin and transferrin, which have both 3'- and 6'-sialyl-*N*-acetyllactosamine terminal structures (Fig 6).

### Validation and characterization of sialyllactose binding

**Fig 7:**
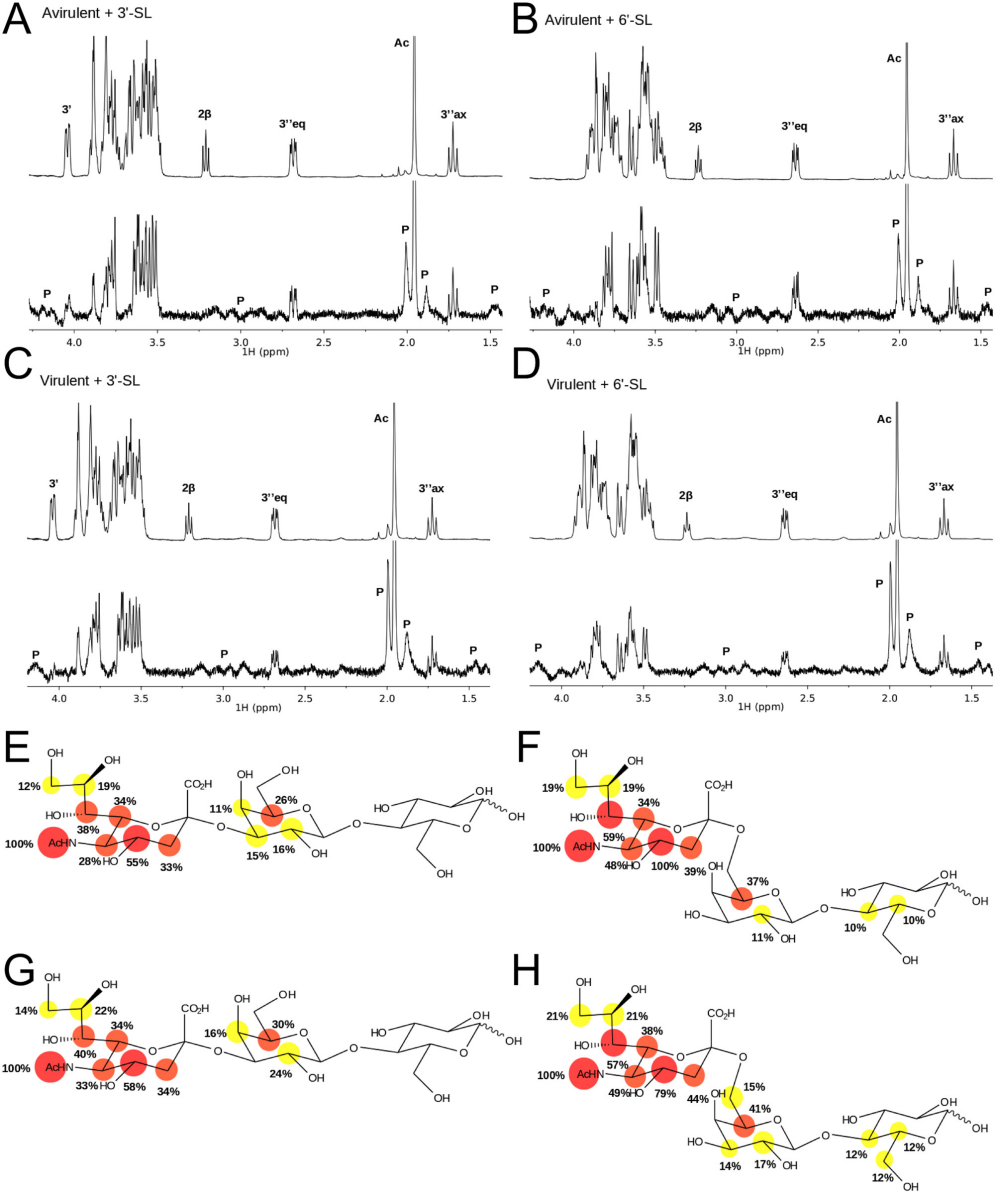
Sialyllactose binding to the TAdV-3 fibre head. Saturation transfer difference NMR experiments performed on 3'- and 6'-sialyllactose in the presence of the avirulent and virulent variants of TAdV-3 fibre head are shown. In panels A through D, the off-resonance reference spectrum (top), with labels indicating the assignment for a number of representative ligand signals, and the STD spectrum (bottom, up-scaled 100X) are shown. Peaks marked with P correspond to signals belonging to the protein. In panels E through H, the epitopes deduced from the STD data are shown, mapped onto the chemical structure of 3'- and 6'-sialyllactose, with labels indicating the STD intensity for each signal, relative to the STD intensity for the 5''-*N*-acetyl peak. Red circles: I > 50%, orange circles: 50% > I > 30%, yellow circles: 30% > I > 10%. A) and E) avirulent variant with 3'-sialyllactose. B) and F) avirulent variant with 6'-sialyllactose. C) and G) virulent variant with 3'-sialyllactose. D) and H) virulent variant with 6'-sialyllactose.

To validate the identified ligand binding, TAdV-3 fibre head protein binding to 3'- and 6'-sialyllactose was examined by saturation transfer difference nuclear magnetic resonance spectroscopy (STD-NMR). The STD experiment is based on the transfer of magnetization between the protein, which is selectively irradiated by a train of radiofrequency pulses, and any binding ligands [77]. For complex ligands, such as oligosaccharides, those atoms lying closer in space to the protein surface receive a higher amount of magnetization, providing information on the ligand contact atoms [78]. When the carbohydrates 3'- and 6'-sialyllactose were incubated in the presence of either the virulent or the avirulent variants of TAdV-3 fibre head proteins, saturation transfer was observed (Fig 7A), which indicated that both ligands interacted with the proteins in solution. In all instances, the highest degree of saturation was observed at the sialic acid, and to a much lesser degree, at the galactose region, and almost none at the glucose (Fig 7B). These experiments not only confirmed the protein-carbohydrate interaction but also mapped the protons of the carbohydrate residues with which the proteins interact, indicating that binding of sialyllactose to TAdV-3 fibre head occurs mainly at the sialic acid residue. STD-NMR was also performed with xylose and, although interaction signals were observed in glycan profiling, no interaction with the proteins could be measured by STD-NMR. The chemical properties of slide surfaces in glycan microarrays as well as different linkers attaching glycans to their carrier molecules can affect the way a glycan is being presented and can impact target protein-ligand interaction [79]. We speculate that this disagreement between glycan microarray profiling and STD-NMR results may be due to the presentation of the carbohydrates and the linker between xylose and bovine serum albumin. The difference in pH (7.7 for STD-NMR and 7.2 for the glycan micro-arrays) may also cause some of the differences observed.

Isothermal titration calorimetry (ITC) was employed to determine the parameters of 3'- and 6'-sialyllactose binding to avirulent and virulent variants of the TAdV-3 fibre head protein. Using ITC, both proteins were found to bind 3'-sialyllactose and 6'-sialyllactose with mM affinity (Table 2; S1 Fig) at pH 6.0, and somewhat less strongly at pH 7.2. The variation of the heat released per mole of ligand injected with the sugar/protein ratio can be described with a model of one binding site per protein monomer subunit. The thermodynamic parameters derived from curve fitting suggested slightly different ways of interaction for the avirulent and virulent forms of the TAdV-3 fibre head protein, since both the enthalpic and the entropic contributions of sugar binding differ. The lower enthalpic stabilization of the complex in the virulent form is partially compensated by a more favourable entropic contribution. Both proteins bound 3'-sialyllactose with two- to four-fold higher affinity than 6'-sialyllactose, while the avirulent form of the protein bound sialyllactose more strongly than the virulent form (1.5- to 3-fold).

**Table 2:** Thermodynamic parameters for 3'- and 6'-sialyllactose binding to TAdV-3 fibre head. Measurements were fitted assuming three independent sites per protein trimer.

Protein	Kd (mM)	- ΔH (kcal/mol of sites)	- ΔS (cal/mol of sites/deg)
	**3'-sialyllactose**		
TAdV-3 (avirulent)^a^	3.64 ± 0.01	4.43 ± 0.01	3.70 ± 0.03
TAdV-3 (virulent)^a^	5.5 ± 0.1	1.83 ± 0.03	-4.19 ± 0.07
R368A^a^,^c^	3.23 ± 0.03	3.86 ± 0.02	1.54 ± 0.05
E392A^a^,^c^	∼ 20	n.d.^c^	n.d.
N407A^a^,^c^	2.77 ± 0.04	4.66 ± 0.04	3.9 ± 0.1
K421A^a^,^c^	n.o.^d^	-	-
K439A^a^,^c^	3.2 ± 0.2	3.9 ± 0.1	1.7 ± 0.2
TAdV-3 (avirulent)^b^	∼24	n.d.^e^	n.d.
TAdV-3 (virulent)^b^	∼37	n.d.^e^	n.d.
	**6'-sialyllactose**		
TAdV-3^a^ (avirulent)	∼7	n.d.^e^	n.d.
TAdV-3^a^ (virulent)	∼20	n.d.^e^	n.d.
TAdV-3^b^ (avirulent)	∼23	n.d.^e^	n.d.
TAdV-3^b^ (virulent)	∼28	n.d.^e^	n.d.

^a^Measurements at pH 6.0.

^b^Measurements at pH 7.2

^c^TAdV-3 fiber head mutants, made in the avirulent context.

^d^no binding observed.

^e^could not be accurately determined.

The STD-NMR experiments were performed in phosphate buffer, pH 7.7, ITC experiments in MES buffer, pH 6.0 and Tris buffer pH 7.2, and glycan microarray profiling in TBS buffer, pH 7.2. The ITC experiments revealed that the TAdV-3 fibre head protein favoured binding to 3'-sialyllactose over 6'-sialyllactose, but a minor preference for 6'-sialyllactose was observed in the glycan microarray platform. These differences could be attributable to the small conformational differences of both protein and carbohydrate ligands in the different buffer systems; and the different ways of presentation (bound to a surface in the glycan micro-arrays, and in solution in the STD-NMR and ITC experiments). Rather than trying to explain these relative differences, we would like to stress the fact that binding of sialyllactose by the TAdV-3 fibre head was observed by all three techniques.

### Sialyllactose binding site on the TAdV-3 fibre head

**Fig 8:**
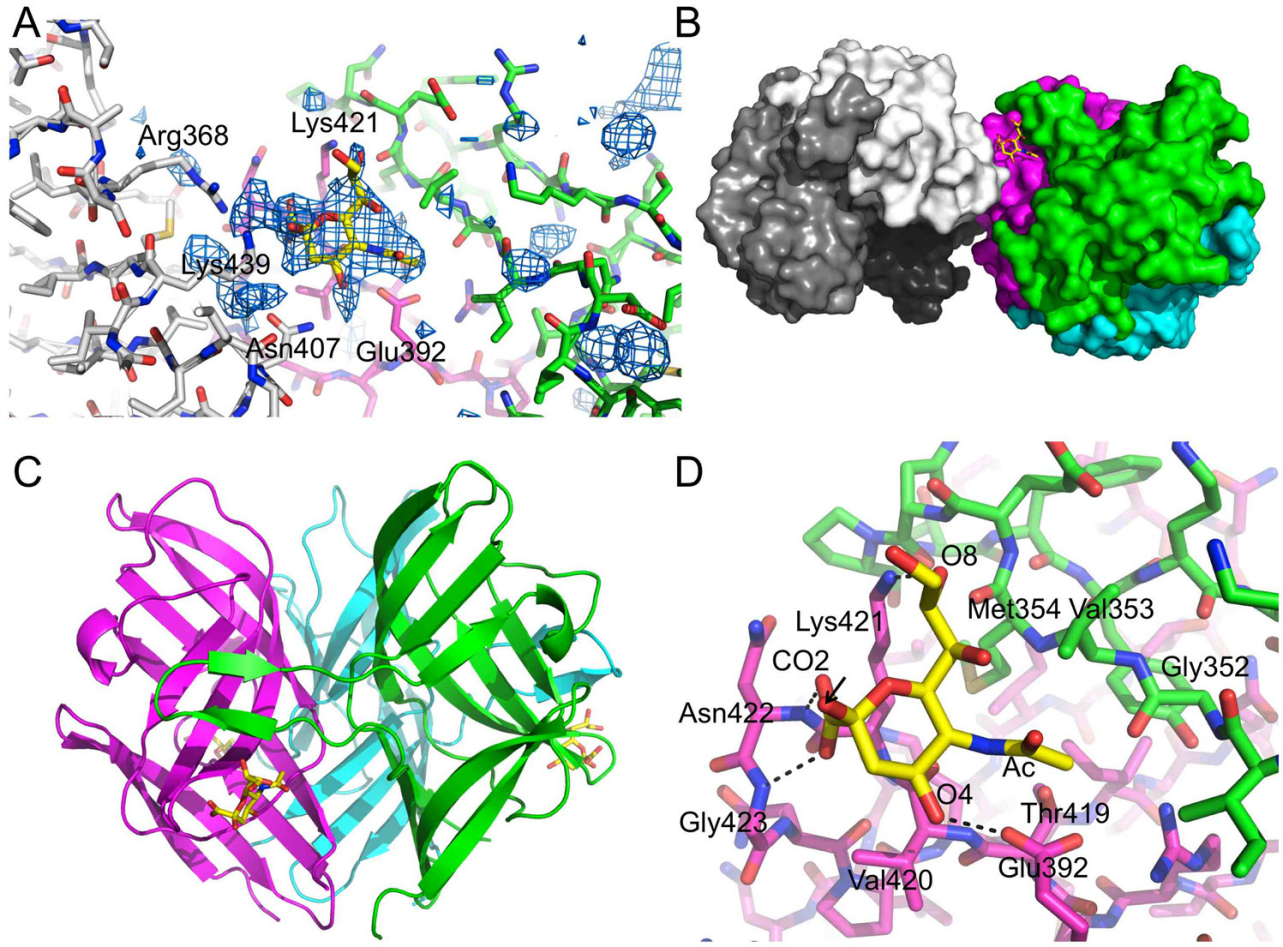
Structure of the TAdV-3 fibre head protein (avirulent form) bound to 3'-sialyllactose. A) Difference density for 3'-sialyllactose obtained after refining the protein model only, before introducing solvent or the ligand, contoured at 2.5 sigma. The main interactions of the sialyllactose are with the magenta monomer shown towards the back. The beta-hairpin from the neighbouring monomer (of the same trimer) is shown in green. A monomer of a neighbouring trimer that approaches the sialyllactose closely in the crystal structure is shown in white. Residues that were selected for site-directed mutagenesis are labelled. B) Location of the ligand between two trimers in the crystal structure. The ligand is shown in yellow, the TAdV-3 fibre head trimer in magenta, green and cyan, and the neighbouring trimer in white, gray and black. In panels C and D, the refined structure of the complex is shown. C) The TAdV-3 fibre head trimer is shown in cartoon representation, in the same colour scheme and orientation as in Fig 1, while sialyllactose ligand is shown in stick representation (carbons yellow). Fig 8C is in the same orientation as the electrostatic representation in Fig 5A. D) Close-up of the binding site for sialyllactose on the fibre head. Interacting residues and relevant atoms of the ligand are labelled (Ac, acetyl; CO2, carboxy). Potential hydrogen bonds are indicated. An arrow indicates the oxygen atom to which the lactose group should be attached. The orientations of panels A, B, and D are the same for clarity.

To obtain information on the binding site of sialyllactose, we soaked crystals of both the avirulent and virulent forms of the TAdV-3 fibre head with 3'- and 6'-sialyllactose. Electron difference density compatible with the sialyl group of the carbohydrate ligand was observed between two trimers in the crystal lattice in soaking experiments with the avirulent version with both 3'- and 6'-sialyllactose (Fig 8). The density for 3'-sialyllactose was stronger than that for 6'-sialyllactose. Density for the lactose group was absent and this part of the ligand was not modelled. In crystals of the virulent variant soaked with either of the two sialyllactoses, some positive electron difference density was also obtained, but not convincing enough to allow modelling.

**Fig 9:**
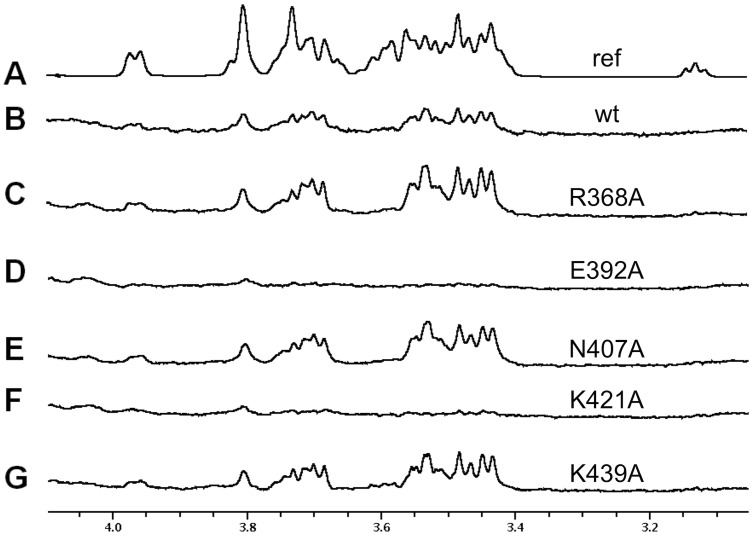
Saturation transfer difference NMR experiments performed on 3'-sialyllactose in the presence of different TAdV-3 fibre head mutants. A) Off-resonance (reference) spectrum. B) STD spectrum with wild-type virulent protein. C) STD spectrum with R368A mutant protein. D) STD spectrum with E392A mutant protein. E) STD spectrum with N407A mutant protein. F) STD spectrum with K421A mutant protein. G) STD spectrum with K439A mutant protein.

Due to the binding of the ligand between two trimers in the crystal lattice, two potential binding sites were identified. The first potential binding site involves residues Glu392, Thr419, Val420, Lys421 and Asn422, while the other potential binding site involves residues Arg368, Asn407 and Lys439. To discriminate between the two possible binding sites, five independent point mutations were introduced, the resulting proteins were expressed and their capacity for binding sialyllactose was analyzed by ITC. Substitution of residues Arg368, Asn407 or Lys439 by alanine had no significant impact on the binding affinity (Table 2). In contrast, substitution of Glu392 by alanine drastically reduced the affinity of the mutant for 3'-sialyllactose and a Lys421Ala mutant rendered 3'-sialyllactose binding undetectable. This indicates that sialyllactose binds to the first site, and not to the second. The orientation of the sialyllactose is consistent with the STD-NMR results: the protons that show most saturation transfer (Fig 7) are closest to the protein. The same mutant proteins were also analyzed by STD-NMR, with the same results, i.e. no effect for the site 2 mutants and strong effects for the site 1 mutants analyzed (Fig 9).

The ligand binding site of the TAdV-3 fibre head is located towards the lower half of the trimeric assembly, away from the 3-fold axis and is constituted by residues Glu392, Thr419, Val420, Lys421, Asn422 and Gly423 from one monomer, and residues Gly352, Val353 and Met354 from the beta-hairpin of the neighbouring monomer (Fig 8). These residues are located on the DG- and HI-loops and on the H-strand of the protein; and on the C'-strand of a neighbouring monomer. The sugar ring of the terminal sialic acid is oriented towards the protein and faces residues Thr419 to Gly423. The carboxylate group of Glu392 interacts with O4 of the sialic acid moiety and, together with the side chain of Thr419, makes van der Waals contact with the acetyl group. Val420 is near the C4 and O4 atoms of the sialic acid, while the amino group of Lys421 contacts O8. The carboxylate group of the sialic acid interacts with the main chain amides of Asn422 and Gly423. From the beta-hairpin of the neighbouring monomer, the carbonyl oxygen of Gly352 is in van der Waals contact with acetyl group of the ligand, while the side-chain of Val353 and the carboxyl oxygen of Met354 contact O8. The side-chain of Met354 faces away from the ligand. Hydrogen bonds formed between Lys421 and carboxylic groups of the ligand appear crucial for TAdV/3'-sialyllactose interaction, as are the interactions between Glu392 and the acetyl part of the ligand. Mutation of these two residues abolishes TAdV/3'-sialyllactose interaction, as described earlier. There were no significant differences between the ligand-free and ligand-bound models and overall r.m.s.d. values of 0.3–0.4 Å were obtained upon superposing them.

Fibre heads of species *Human mastadenovirus D* types like HAdV-19 and HAdV-37 have their sialic acid binding sites on the top of the trimer, near the three-fold axis [73]. HAdV-52, from the new species *Human mastadenovirus G*, also has its sialic acid binding site near the top of the trimer, although further away from the central three-fold axis, towards the side of the trimer [80].

### Conclusion

We solved the atomic structure of the fibre head of the avirulent and virulent variants of turkey adenovirus 3 (TAdV-3). This is the first siadenovirus for which the structure of the fibre head has been determined. The structure revealed a positively charged surface, which may be important for host cell interaction. A beta-hairpin insertion in the C-strand contacting a neighbouring monomer is unique to the TAdV-3 structure. The fibre head shares the secondary structure topology with other adenovirus and reovirus fibre heads and with certain bacteriophage baseplate protein receptor-binding domains. Curiously, its closest structural homologues are reovirus fibre heads. The sialyl group of sialyllactose was identified as a possible receptor for the TAdV-3 fibre head and its binding site was delineated. However, the binding affinity is low (mM), although avidity may increase the effective affinity via the three binding sites on the trimer and the virus binding the host cell with multiple fibres simultaneously [81]. The real receptor may well be a more complex carbohydrate or a sialylated cell surface molecule, possibly glycolipid in nature, with a more extensive interaction footprint on the fibre head. Knowledge of the structure and receptor-binding properties of the TAdV-3 fibre head may allow the design of chimeric adenoviruses incorporating the TAdV-3 fibre head and investigating their tropism, which in turn may lead to vaccination or gene therapy vectors that may target specific cell types.

## Supporting Information

S1 Fig ITC titration profile of avirulent (left) and virulent (right) TAdV-3 protein with 3'-sialyllactose in buffer containing 10 mM MES-NaOH, pH 6.0, performed at 25°C. Top panels show the experimental curves and bottom panels the dependence of heat evolved per mole of the injected ligand with the ligand/protein molar ratio. The continuous line is the fit of a one-site model per monomer to the experimental data.
(TIFF)

S1 Table Neoglucoconjugates and glycoproteins used in the glycan microarray. Names of the neoglucoconjugates and glycoproteins, print concentrations, explanation of abbreviations and chemical structures are shown.
(PDF)
