# Supplementary material for: Structure and Sialyllactose Binding of the Carboxy-Terminal Head Domain of the Fibre from a Siadenovirus, Turkey Adenovirus 3
Source: PLoS One. 2015 Sep 29;10(9):e0139339. doi: 10.1371/journal.pone.0139339 (PMC4587935; doi:10.1371/journal.pone.0139339)
Supplement: S1 Table — Names of the neoglucoconjugates and glycoproteins, print concentrations, explanation of abbreviations and chemical structures are shown. (PDF) [file pone.0139339.s002.pdf]

| | Abbreviation | Neoglycoconjugate/ glycoprotein | Print conc (mg/mL) | Structure |
|---|---|---|---|---|
| 1 | Fetuin | Fetuin | 1 | Bovine fetuin |
| 2 | ASF | Asialofetuin | 1 | Bovine asialofetuin |
| 3 | PBS | PBS | 1 | Phosphate buffered saline |
| 4 | Ov | Ovalbumin | 1 | Hen ovalbumin |
| 5 | RB | RNAse B | 1 | Ribonuclease B |
| 6 | Xferrin | Transferrin | 1 | Bovine transferrin |
| 7 | 4APHSA | 4AP-HSA | 1 | 4AP-HSA, linker alone attached to HSA |
| 8 | α-C | α-Crystallin from bovine lens | 1 | α-Crystallin from bovine lens, A and B subunits |
| 9 | M3BSA | Manα1,3(Manα1,6)Man-BSA | 1 | Man-α–(1,3)-[Man-α–(1,6)-]Man-BSA |
| 10 | GlcNAcBSA | GlcNAc-BSA | 1 | GlcNAc-Sp14-NH2(Lys)-BSA |
| 11 | LacNAcBSA | LacNAc-BSA | 1 | Gal-β-(1,4)-GlcNAc-Sp3-BSA |
| 12 | 3SLNBSA | 3'SialylLacNAc-BSA | 1 | Neu5Ac-α-(2,3)-Gal-β-(1,4)-GlcNAc-BSA |
| 13 | 3SLacHSA | 3´-Sialyllactose-APD-HSA | 1 | Neu5Ac-α-(2,3)-Gal-β-(1,4)-Glc-APD-HSA |
| 14 | 6SLacHSA | 6´-Sialyllactose-APD-HSA | 1 | Neu5Ac-α-(2,6)-Gal-β-(1,4)-Glc-APD-HSA |
| 15 | 2FLBSA | 2'Fucosyllactose-BSA | 1 | Fuc-α-(1,2)-Gal-β-(1,4)-Glc-Sp3-BSA |
| 16 | 3SFLBSA | 3'Sialyl-3-fucosyllactose-BSA | 1 | Neu5Ac-α-(2,3)-Gal-β-(1,4)-[Fuc-α-(1,3)-]Glc-Sp3-BSA |
| 17 | H2BSA | H Type II-APE-BSA | 1 | Fuc-α-(1,2)-Gal-β-(1,4)-GlcNAc-β-APE-BSA |
| 18 | BGABSA | Blood Group A-BSA | 1 | GalNAc-α-(1,3)-[Fuc-α-(1,2)-]Gal-β-(1,4)-GlcNAc-Sp6-BSA |
| 19 | BGBBSA | Blood Group B-BSA | 1 | Gal-α-(1,3)-[Fuc-α-(1,2)-]Gal-β-(1,4)-GlcNAc-Sp6-BSA |
| 20 | GGGNHSA | Galα1,3Galβ1,4GlcNAc-HSA | 1 | Gal-α–(1,3)-Gal-β–(1,4)-GlcNAc-HSA |
| 21 | Ga3GBSA | Galα1,3Gal-BSA | 1 | Gal-α–(1,3)-Gal-Sp3-BSA |
| 22 | Gb4GBSA | Galb1,4GalBSA | 1 | Gal-β-(1,4)-Gal-Sp3-BSA |
| 23 | Ga2GBSA | Gala1,2GalBSA | 1 | Gal-α–(1,2)-Gal-Sp3-BSA |
| 24 | 4APBSA | 4AP-BSA | 1 | 4AP-BSA, linker alone attached to BSA |
| 25 | LNFPIBSA | Lacto-*N*-fucopentaose I-BSA | 1 | Fuc-α-(1,2)-Gal-β-(1,3)-GlcNAc-β-(1,3)-Gal-β-(1,4)-Glc-BSA |
| 26 | LNFPIIBSA | Lacto-*N*-fucopentaose II-BSA | 1 | Fuc-α-(1,3)-Gal-β-(1,3)-GlcNAc-β-(1,3)-Gal-β-(1,4)-Glc-BSA |
| 27 | LNFPIIIBSA | Lacto-*N*-fucopentaose III-BSA | 1 | Gal-β-(1,4)-[Fuc-α-(1,3)-]GlcNAc-β-(1,3)-Gal-β-(1,4)-Glc-BSA |
| 28 | LNDHIBSA | Lacto-*N*-difucohexaose I-BSA | 1 | Fuc-a-(1,2)-Gal-b-(1,3)-[Fuc-a-(1,4)-]GlcNAc-b-(1,3)-Gal-b-(1,4)-Glc-Sp3-BSA |
| 29 | LebBSA | LNDI-BSA/ Lewis b-BSA | 1 | Fuc-α-(1,2)-Gal-β-(1,3)-[Fuc-α-(1,4)-]GlcNAc-β-(1,3)-Gal-β-(1,4)-Glc-APD-BSA |
| 30 | LexBSA | Lewis x-BSA | 1 | Gal-β-(1,4)-[Fuc-α-(1,3)-]GlcNAc-BSA |
| 31 | DiLexBSA | Di-Lex-APE-BSA | 1 | Gal-β-(1,4)-[Fuc-α-(1,3)-]GlcNAc-β-(1,3)-Gal-β-(1,4)-[Fuc-α-(1,3)-]GlcNAc-β-O-APE-BSA |
| 32 | DiLexHSA | Di-Lewisx-APE-HSA | 1 | Gal-β-(1,4)-[Fuc-α-(1,3)-]GlcNAc-β-(1,3)-Gal-β-(1,4)-[Fuc-α-(1,3)-]GlcNAc-β-O-APE-HSA |
| 33 | 3LexHSA | Tri-Lex-APE-HSA | 1 | Gal-β-(1,4)-[Fuc-α-(1,3)-]GlcNAc-β-(1,3)-Gal-β-(1,4)-[Fuc-α-(1,3)-]GlcNAc-β-(1,3)-Gal-β-(1,4)-[Fuc-α-(1,3)-]GlcNAc-β-O-APE-HSA |
| 34 | 3SLexBSA3 | 3'Sialyl Lewis x-BSA | 1 | Neu5Ac-α-(2,3)-Gal-β-(1,4)-[Fuc-α-(1,3)-]GlcNAc-Sp3-BSA |
| 35 | SLexBSA14 | 3'Sialyl Lewis x-BSA | 1 | Neu5Ac-α-(2,3)-Gal-β-(1,4)-[Fuc-α-(1,3)-]GlcNAc-Sp14-BSA |
| 36 | 6SuLexBSA | 6-Sulfo Lewis x-BSA | 1 | (SO4)6Gal-β-(1,4)-[Fuc-α-(1,3)-]GlcNAc-Sp3-BSA |
| 37 | 6SuLeaBSA | 6-Sulfo Lewis a-BSA | 1 | (SO4)6Gal-β-(1,3)-[Fuc-α-(1,4)-]GlcNAc-Sp3-BSA |
| 38 | 3SuLeaBSA | 3-Sulfo Lewis a-BSA | 1 | (SO4)3Gal-β-1-3-[Fuc-α-(1,4)-]GlcNAc-Sp3-BSA |
| 39 | 3SuLexBSA | 3-Sulfo Lewis x-BSA | 1 | (SO4)3Gal-β-1-4-[Fuc-α-(1,3)-]GlcNAc-Sp3-BSA |
| 40 | DFPLNHHSA | Difucosyl-para-lacto-N-hexaose-APD-HSA, (Lea/Lex) | 1 | Gal-β-(1,3)-[Fuc-α-(1,4)-]GlcNAc-β-(1,3)-Gal-β-(1,4)-[Fuc-α-(1,3)-]GlcNAc-β-(1,3)-Gal-β-(1,4)-Glc-APD-HSA |
| 41 | LeyHSA | Lewis y-tetrasaccharide-APE-HSA | 1 | Fuc-α-(1,2)-Gal-β-(1,4)-[Fuc-α-(1,3)-]GlcNAc-β-O-APE-HSA |
| 42 | 3FLeyHSA | Tri-fucosyl-Ley-heptasaccharide-APE-HSA | 1 | Fuc-α-(1,2)-Gal-β-(1,4)-[Fuc-α-(1,3)-]GlcNAc-β-(1,3)-Gal-β-(1,4)-[Fuc-α-(1,3)-]GlcNAc-β-O-APE-HSA |
| 43 | LNnTHSA | Lacto-N-neotetraose-APD-HSA | 1 | Gal-β-(1,4)-GlcNAc-β-(1,3)-Gal-β-(1,4)-Glc-APD-HSA |
| 44 | LNTHSA | Lacto-N-tetraose-APD-HSA | 1 | Gal-β-(1,3)-GlcNAc-β-(1,3)-Gal-β-(1,4)-Glc-APD-HSA |
| 45 | SLNFVHSA | Sialyl-LNF V-APD-HSA | 1 | Fuc-α-(1,2)-Gal-β-(1,3)-[NeuAc-α-(2,6)-]GlcNAc-β-(1,3)-Gal-β-(1,4)-Glc-APD-HSA |
| 46 | MMLNnHHSA | Monofucosyl, monosialyllacto-N-neohexaose-APD-HSA | 1 | Neu5Ac-α-(2,3)-Gal-β-(1,4)-GlcNAc-β-(1,3)-[Gal-β-(1,4)-[Fuc-α-(1,3)-]GlcNAc-β-(1,6)-]Gal-β-(1,4)-Glc-APD-HSA |
| 47 | SLNnTHSA | Sialyl-LNnT-penta-APD-HSA | 1 | Neu5Ac-α-(2,3)-Gal-β-(1,4)-GlcNAc-β-(1,3)-Gal-β-(1,4)-Glc-APD-HSA |
| 48 | GM1HSA | GM1-pentasaccharide-APD-HSA | 1 | Gal-β-(1,3)-GalNAc-β-(1,4)-[Neu5Ac-α-(2,3)-]Gal-β-(1,4)-Glc-APD-HSA |
| 49 | aGM1HSA | Asialo-GM1-tetrasaccharide-APD-HSA | 1 | Gal-β-(1,3)-GalNAc-β-(1,4)-Gal-β-(1,4)-Glc-APD-HSA |
| 50 | GlobNTHSA | Globo-N-tetraose-APD-HSA | 1 | GalNAc-β-(1,3)-Gal-α-(1,4)-Gal-β-(1,4)-Glc-APD-HSA |
| 51 | GlobTHSA | Globotriose-APE-HSA | 1 | Gal-α-(1,4)-Gal-β-(1,4)-Glc-β-APE-HSA |
| 52 | Inv | Invertase | 1 | Yeast invertase, grade VII |
| 53 | Fibrin | Fibrinogen | 0.5 | Fibrinogen from human plasma |
| 54 | A1AT | alpha-1-antitrypsin | 1 | alpha-1-antitrypsin |
| 55 | Cerulo | Ceruloplasmin | 1 | Ceruloplasmin, human, type III |
| 56 | AGP | alpha-1-acid glycoprotein | 1 | alpha-1-acid glycoprotein, human |
| 57 | LacNAcaBSA | LacNAc-a-4AP-BSA | 1 | Gal-β-(1,4)-GlcNAc-α-4AP-BSA |
| 58 | LacNAcb4APBSA | LacNAc-b-4AP-BSA | 1 | Gal-β-(1,4)-GlcNAc-β-4AP-BSA |
| 59 | H2HSA | H-Type 2-APE-HSA | 1 | Fuc-α-(1,2)-Gal-β-(1,4)-GlcNAc-β-APE-HSA |
| 60 | Ovomuc | Ovomucoid | 0.5 | Partially purified ovomucoid, chicken |
| 61 | RhaBSA | L-Rhamnose-Sp14-BSA | 1 | L-Rhamnose-Sp14-BSA |
| 62 | XLacbBSA | Lac-β-4AP-BSA | 1 | Lac-β-4AP-BSA |
| 63 | XylbBSA | Xyl-β-4AP-BSA | 1 | Xyl-β-4AP-BSA |
| 64 | XylaBSA | Xyl-α-4AP-BSA | 1 | Xyl-α-4AP-BSA |
| 65 | XGlcbBSA | Glc-β-4AP-BSA | 1 | Glc-β-4AP-BSA |
| 66 | FucaBSA | Fuc-α-4AP-BSA | 1 | Fuc-α-4AP-BSA |
| 67 | FucbBSA | Fuc-β-4AP-BSA | 1 | Fuc-β-4AP-BSA |
| 68 | Galb4APBSA | Gal-b-4AP-BSA | 1 | Gal-b-4AP-BSA |
| 69 | Neu5GcBSA | Neu5Gc-α-4AP-BSA | 1 | Neu5Gc-α-4AP-BSA |
| 70 | CollagenIV | Collagen type IV | 0.25 | Collagen type IV |
| 71 | D-GlobTHSA | Globotriose-HSA | 1 | Gal-α-(1,4)-Gal-β-(1,4)-Glc-Sp3-BSA |

**Linker key**

Sp3 = 3 atom spacer
Sp6 = 6 atom spacer
Sp14 = 14 atom linker
4AP = 4-aminophenyl
APE = aminophenylethyl
APD = acetylphenylenediamine
